# Pharmacophoric Site Identification and Inhibitor Design for Autotaxin

**DOI:** 10.3390/molecules24152808

**Published:** 2019-08-01

**Authors:** Myeong Hwi Lee, Dae-Yon Lee, Anand Balupuri, Jong-Woo Jeong, Nam Sook Kang

**Affiliations:** 1 Graduate School of New Drug Discovery and Development, Chungnam National University, 99 Daehak-ro, Yuseong-gu, Daejeon 34134, Korea; 2 LegoChem Biosciences, Inc., 8-26 Munoyeongseo-ro, Daedeok-gu, Daejeon 34302, Korea

**Keywords:** autotaxin, topological water networks, molecular docking, molecular dynamics simulation

## Abstract

Autotaxin (ATX) is a potential drug target that is associated with inflammatory diseases and various cancers. In our previous studies, we have designed several inhibitors targeting ATX using computational and experimental approaches. Here, we have analyzed topological water networks (TWNs) in the binding pocket of ATX. TWN analysis revealed a pharmacophoric site inside the pocket. We designed and synthesized compounds considering the identified pharmacophoric site. Furthermore, we performed biological experiments to determine their ATX inhibitory activities. High potency of the designed compounds supports the predictions of the TWN analysis.

## 1. Introduction

Autotaxin (ATX) is an enzyme that hydrolyzes lysophatidylcholine (LPC) to lysophosphatidic acid (LPA) in the extracellular matrix [1,2]. The LPA produced in the process binds to the LPA receptors. This leads to a number of signal transduction processes such as cell proliferation, cell survival, cell migration and secretion of cytokines and chemokines [3,4]. These signal transduction processes are associated with several pathological conditions such as angiogenesis, chronic inflammation, fibrosis, metastasis and various cancers. The LPA-related signal transduction processes have been actively studied in the inflammatory diseases including asthma, arthritis, fibrosis of the lungs, liver and skin and various cancers [5,6,7,8,9]. Several ATX inhibitors have been reported up till now [10,11,12] but no ATX inhibitor has been approved for treatment yet. GLPG-1690 reported by Galapagos NV [13] is merely one ATX inhibitor that is under clinical trials for the treatment of idiopathic pulmonary fibrosis. This compound has completed Phase Ⅰ (ClinicalTrials.gov Identifiers: NCT02738801, NCT03143712, NCT02179502, and NCT03515382) and Phase Ⅱ (ClinicalTrials.gov Identifier: NCT02738801) clinical trials. It is currently in Phase Ⅲ clinical trials (ClinicalTrials.gov Identifiers: NCT03733444 and NCT03711162).

Proteins in the biological environment are surrounded by water molecules. They play an important role in the structure, stability, folding, dynamics and function of proteins [14,15]. Water molecules present in different sites of biomolecules establish hydrogen bonds with the proteins and contribute to the stability of their three-dimensional structures [14]. Thermodynamic changes in the aqueous environment affect the stability of biomolecules. Water molecules contribute to the catalytic function of proteins and their dynamics [15,16]. They control protein folding through hydrophobic collapse [15]. Binding sites of the proteins in the apo condition are filled with several water molecules that play an important role in the recognition of the ligands [17,18]. They effect the binding process between proteins and small molecule ligands [19,20,21,22]. Water molecules can be displaced by the ligands or they may act as bridges to stabilize the formation of a complex [23,24,25]. Displacement of water molecules from the active site to the bulk region occurs when a ligand binds to a protein. The thermodynamics of this displacement process is a main source of binding free energy of ligands [26,27,28]. Water molecules mediate the interactions between the ligand and the protein and form hydrogen-bonded networks that stabilize a protein−ligand complex. Water molecules also influence the unbinding of ligands from host substrates [29], and dissociation of biological complexes [30]. Besides, they effect the aggregation of amyloid proteins [31,32,33]. Previously, not much emphasis was given on the role of water in the binding process and drug discovery. However, water molecules have recently attained the deserved attention in the discovery and development of the drugs [34,35]. These days, the displacing, mimicking, and/or targeting of bound water molecules is carried out in the structure-based drug design to improve the binding affinity of ligand molecules [36,37]. The accuracy of protein–ligand docking in the presence of tightly bound water molecules was evaluated. Crystallographic water molecules improved the docking accuracy [26,37,38]. They also improved the accuracy of virtual screening. Water molecules aided in the placement of ligands close to the center of the active sites, besides mediating the interactions between ligands and proteins [37,38].

Water molecules form cyclic structures through hydrogen bonding. We investigated the tendency of water molecules to form three to six membered rings earlier and termed this exploration as topological water network (TWN) analysis [33,39,40,41,42]. We used TWN analysis to explain kinase selectivity [39,40,41], to examine protein–ligand binding [40], to explore protein folding [33] and to investigate protein hydration [42] in our former studies. Most importantly, we developed a selective interleukin-1 receptor-associated kinase 4 (IRAK4) inhibitor using this approach [41]. Our research group has also reported potent ATX inhibitors in our previous papers [43,44]. In the present study, we carried out molecular dynamics (MD) simulation on the ATX structure in the apo state. The TWN analysis was performed around an ATX inhibitor PF-8380 that was reported by Pfizer Inc. [45]. This compound was selected as a template due to its high potency and its known binding mode. Novel compounds were designed and synthesized to investigate the TWN results. Furthermore, biological activities of the compounds were measured using the in vitro bis-pNPP [46] and ex vivo human plasma assays.

## 2. Results and Discussion

### 2.1. TWN Analysis and Identification of the Pharmacophoric Sites

**Figure 1:**
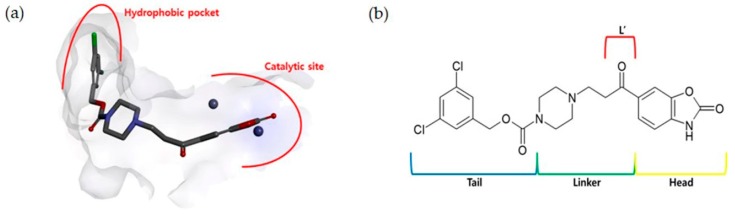
(**a**) A simplified schematic binding mode of PF-8380 (gray stick model) inside the autotaxin (ATX) binding pocket. Two zinc atoms in the catalytic site of ATX are represented by dark blue spheres. (**b**) 2D structure of PF-8380 divided into head, linker and tail regions. The CO group of the linker region is marked with L’.

ATX is composed of somatomedin B (SMB) like domains, a catalytic phosphodiesterase domain (PDE) and a C-terminal nuclease-like domain (NUC) [47]. In the present study, we have targeted the catalytic domain. This domain consists of a catalytic site for hydrolytic activity with conserved zinc ions, a hydrophobic pocket, a hydrophobic tunnel and a T-shaped structure [11,12,48,49]. Previously, we designed ATX inhibitors targeting the catalytic site and the hydrophobic pocket [43,44]. These compounds exhibited a binding mode similar to the known ATX inhibitors such as 3BoA from the University of Tokyo [50] and PF-8380 from Pfizer Inc. [45]. As explained in the methodology section, we carried out MD simulation on the crystal structure of ATX (PDB ID 3WAX) [50] in the apo state. A highly potent ATX inhibitor (PF-8380, IC_50_ = 2.80 nM) [45] was placed in the binding site and TWN analysis was performed around it. Binding mode of PF-8380 is known [45]. As shown in Figure 1a, it occupies the catalytic site and the hydrophobic pocket. A Two-dimensional (2D) structure of the PF-8380 structure was divided into head, linker and tail regions (Figure 1b) for TWN analysis. The above-mentioned protein structure and PF-8380 were used as the reference protein and ligand, respectively, in this study.

**Figure 2:**
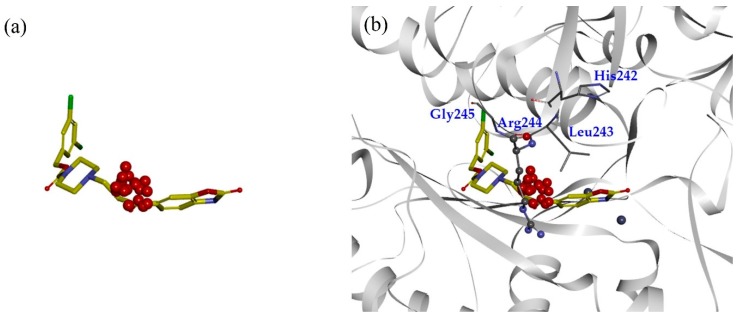
(**a**) Structure of PF-8380 overlapped with topological water networks (TWNs) of the L’ region. (**b**) Overlay of ligand and TWNs in the ATX binding site. Centers of mass of water networks are represented by red spheres while PF-8380 is displayed by gray stick model. Binding site residues His242, Leu243 and Gly245 are represented by gray thick line model, whereas the Arg244 that exists adjacent to the L’ region is shown by ball and stick model.

MD simulations and TWN analysis were performed as described in the methodology section. The results showed 35.1% and 64.9% TWNs for the head and linker sites, respectively. Interestingly, most of the water molecules in the linker site (75%) were located near the CO group (L’) at the point where the linker and the head are connected. This suggested that water is always present in that vicinity in any form. In contrast with the head and linker sites, we could not identify the TWNs in the tail site. This site is narrow and the space is limited. Thus, water molecules could not form a ring structure. Moreover, this site lies in the hydrophobic pocket and possesses hydrophobic characteristics. TWNs around the L’ position are represented by red spheres in Figure 2a. As can be seen in Figure 2b, there is a loop consisting of the residues His242, Leu243, Arg244, and Gly245 nearby the L’ position. Arg244 is located at a position where it is capable of forming a hydrogen bond with the ligand.

Additionally, we calculated the shape similarity between the ligand and the water molecules. Similarity in shape is used to compare the geometric characteristics of a ligand and the water molecule networks. Further details and in-house codes for the shape similarity are provided in our previous research paper [41]. Water networks whose centers of mass were located within 2 Å from PF-8380 were extracted for the shape similarity calculation. A similarity of 23.4% was obtained between the ligand and TWNs when the entire ligand structure was considered. However, 73.7% similarity was observed when the analysis was focused on the L’ region. This result also indicated that hydrophilic interactions involving hydrogen bonds at the L’ position could be important for the ATX inhibitory activity.

### 2.2. Design, Synthesis and Activity Evaluation

Based on the TWN analysis, hydrophilic interactions at the L’ position were supposed to be important. To confirm this hypothesis, we designed and synthesized compounds which could form hydrogen bonds with Arg244. Finally, we verified their ATX inhibitory activities through in vitro bis-pNPP assay.

**Figure 3:**
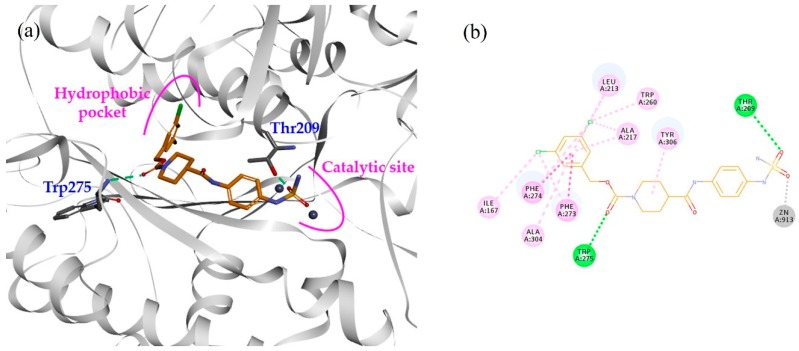
(**a**) Binding mode of compound **1** (orange stick model) inside the ATX binding pocket. It occupies the catalytic site as well as the hydrophobic pocket. (**b**) 2D interaction diagram of compound **1 (**orange line model). Hydrogen bonds and hydrophobic interactions with the active site residues are displayed as green and pink dashed lines, respectively. Electrostatic interaction with catalytic zinc ion is represented by gray dashed line.

**Scheme 1:**
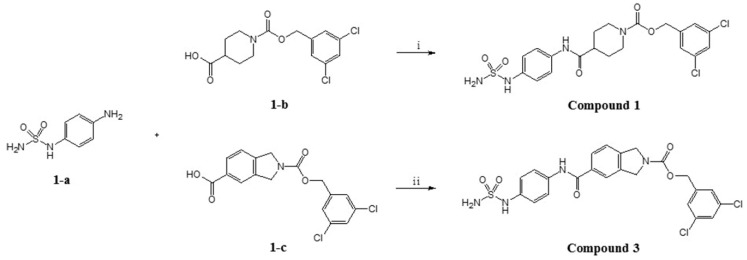
Synthesis of compound **1** and compound **3**. Reagents and conditions. (ⅰ) HOBt, DMF, TEA, EDC, rt. 20 h. (ⅱ) DIPEA, PyBOP, DMF, rt, 15 h.

**Scheme 2:**
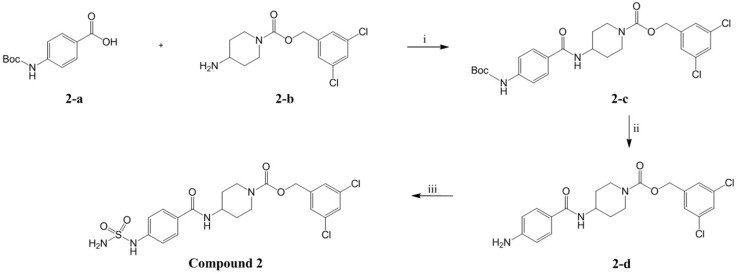
Synthesis of compound **2**. Reagents and conditions. (ⅰ) HOBt, DMF, TEA, EDC, rt, 16 h. (ⅱ) DCM, 4 N HCl, rt, 4 h. (ⅲ) DIPEA, DMF, rt, 18 h.

**Scheme 3:**
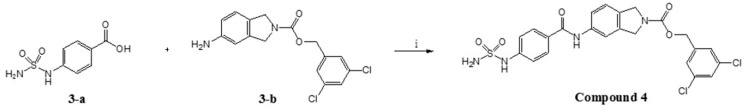
Synthesis of compound **4**. Reagents and conditions. (ⅰ) DIPEA, PyBOP, DMF, rt, 15 h.

In our previous study, compound **1** showed an IC_50_ value of 250.0 nM in the bis-pNPP assay [18]. Probable binding mode for this compound is shown in Figure 3a. It can be seen that head and tail regions of the compound occupy the catalytic site and the hydrophobic pocket, respectively. Both head and tail regions showed hydrogen-bonding interactions, however, no hydrogen bond was observed for the linker region (Figure 3b). Residues Thr209 and Trp275 were involved in hydrogen-bonding interactions with compound **1**. We used compound **1** as a reference for designing compounds **2**–**4**. Structures, IC_50_ values, and predicted physicochemical properties of the designed compounds are summarized in Table 1. The synthesis of the compounds is shown in Scheme 1, Scheme 2 and Scheme 3.

**Figure 4:**
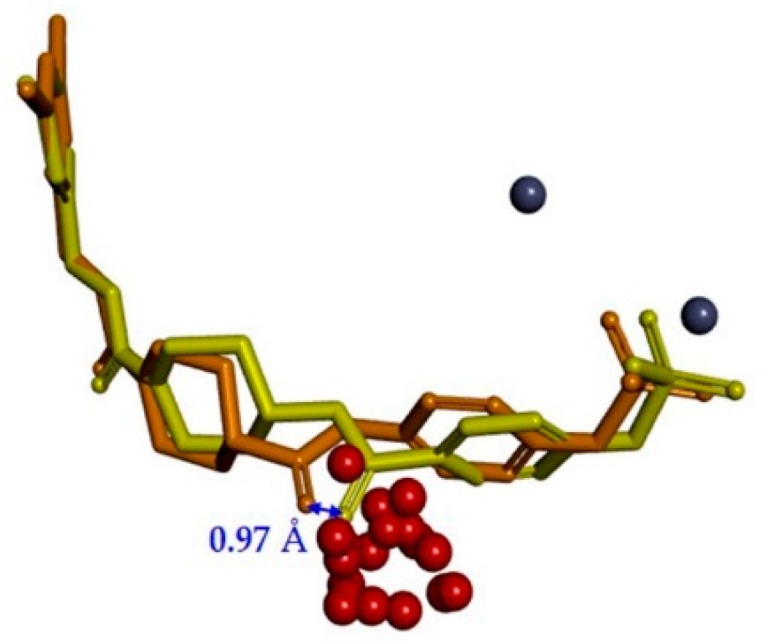
Comparison of the binding modes of compound **1** (orange stick model) and compound **2** (yellow stick model). The distance between CO groups of compound **1** and compound **2** was found to be 0.97 Å. The CO group of compound **2** was located closer to the L’ region. The centers of mass of water networks and zinc atoms are represented by red and dark blue spheres, respectively.

As shown in Figure 3b, the predicted binding mode of compound **1** did not show any hydrogen bond with Arg244. TWN analysis suggested that this interaction could be significant. Accordingly, we interchanged NH and CO positions in compound **2**. In the docking results, the CO group of compound **2** was located closer to the Arg244 as compared to the CO group of compound **1**. It was able to form a hydrogen bond with Arg244. Figure 4 shows a comparison of the binding modes of compounds **1** and **2**. As shown in the figure, CO group of compound **2** was positioned nearer to the TWNs observed around the L’ region. Based on these results, compound **2** was anticipated to be more potent than compound **1**. As expected, compound **2** (IC_50_ = 44.50 nM) was found to be five-fold more potent than compound **1** (IC_50_ = 250.00 nM) in the bis-pNPP assay.

**Figure 5:**
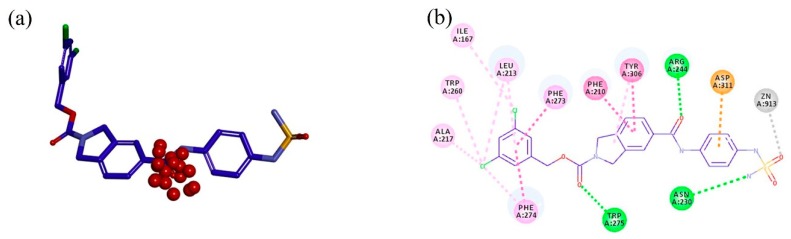
(**a**) Binding mode of compound **3** (cyan stick model) overlapped with TWNs of the L’ region. (**b**) 2D interaction diagram of compound **3** (purple line model). Hydrogen bond, pi-anion and hydrophobic interactions with the active site residues are shown as green, orange and pink dashed lines, respectively. The electrostatic interaction with a catalytic zinc ion is represented by the gray dashed line.

Compound **3** was designed by replacing the piperidine scaffold of compound **1** with a 2,3-dihydro-1H-isoindole moiety. This change produced a difference in the overall length of the linker and thereby affected the position of the linker CO group. The binding mode of compound **3,** predicted by docking, indicated that a new fragment would enable the CO group of the linker to form hydrogen bonds with Arg244. As shown in Figure 5a, the linker CO group of compound **3** overlaps well with the TWNs of the L’ region, and has good orientation towards TWNs. Thus, we believed that compound **3** would be able to form a strong hydrogen bond with Arg244. It can be seen in Figure 5b that compound **3** formed a hydrogen bond with Arg244. Moreover, as expected, compound **3** showed an IC_50_ value of 3.86 nM. In addition, it demonstrated an IC_50_ value of 827.00 nM in the human plasma assay.

**Figure 6:**
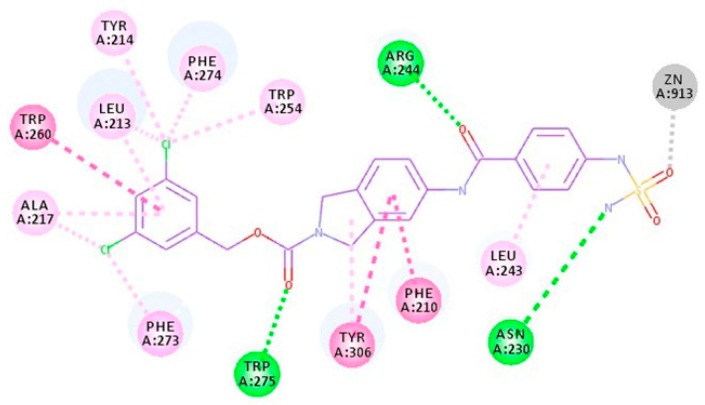
2D interaction diagram of compound **4** (purple line model). Hydrogen bonds and hydrophobic interactions with the active site residues are displayed as green and pink dashed lines, respectively. Electrostatic interaction with catalytic zinc ion is represented by the gray dashed line.

We interchanged NH and CO positions of compound **3** to design compound **4**. As shown in Figure 6, compound **4** was able to form a hydrogen bond with Arg244 in the docking results. However, it may not be as strong as compound **3** due to a slight change in CO position and orientation. Also, docking results suggested a slight change in the interactions of the head region. The head group of compound **3** showed a pi-anion interaction with Asp311, while the head group of compound **4** showed hydrophobic interaction with Leu243. Thus, activity of compound **4** was expected to be slightly lower than compound **3**. As anticipated, activity of compound **4** (IC_50_ = 12.70 nM) was marginally reduced in comparison to the compound **3** (IC_50_ = 3.86 nM).

In addition, we calculated the shape similarity between the designed compounds and the TWNs in the same way as for PF-8380. The shape similarities for compounds **1**, **2**, **3** and **4** considering the whole ligand structure were found to be 22.9%, 21.3%, 17.1%, and 21.6%, respectively. The shape similarities concentrated in the L’ region were 64.1%, 64.1%, 65.5%, and 65.5% for the compounds **1**, **2**, **3** and **4**, respectively. The shape similarity results for the L’ region appear to be very similar. However, the results can be interpreted differently when compared with the similarity obtained for the whole structure. Although compound **3** showed a low similarity of 17.1% for the overall structure, a high similarity of 65.5% was obtained for the analysis focused on the L’ position. Given the lower similarity for the overall structure, the similarity at the L’ position can be considered relatively high, compared to the other compounds. Through this analysis, it can be interpreted that the CO group of compound **3** is in a position where it is properly overlapped with the TWNs.

## 3. Materials and Methods

### 3.1. Protein Preparation

Protein structures were obtained from the RCSB Protein Data Bank (PDB) [51]. Except the two zinc ions, co-crystallized ligand and water molecules were removed from the crystal structure. Missing residues, bond order, and partial charges were checked using the protein preparation protocol of Discovery Studio 2018 (BIOVIA, San Diego, CA, USA). Amino acids were assumed to be in an ionized state at pH 7.4.

### 3.2. MD Simulation

MD simulation was performed using GROMACS 4.5.3 [52]. Topological files were generated using the GROMOS96 43a1 force field [53]. Protein was immersed in a cubic box of SPC/E water model [28] with a margin distance of 10 Å. The system was neutralized by adding 3 Cl^-^ counter ions. Energy minimization was carried out for 500 steps using the steepest descent method. Equilibration was performed to stabilize temperature (300 K) and pressure (1 bar) of the system. Initially, equilibration was conducted at 300 K temperature for 100 ps under NVT ensemble (canonical ensemble). Afterwards, equilibration was conducted at 1 bar pressure for 200 ps under NPT ensemble (isothermal-isobaric ensemble). Subsequently, production run was carried out for 10 ns at 300 K temperature and 1 bar pressure. Coordinate trajectories were saved every 10 ps. Temperature and pressure were regulated by Berendsen thermostat [54] and Parrinello-Rahman barostat [55], respectively. The cutoff for short-range non-bonded interactions was set to 1.2 nm. Long-range electrostatics were calculated by the particle-mesh Ewald method [56]. A linear constraint solver (LINCS) algorithm [57] was used for constraining bond lengths. Periodic boundary conditions were applied in all the dimensions.

### 3.3. TWN Analysis

Water molecules form a number of cyclic water-ring networks through hydrogen-bonding between themselves. These networks are known as TWNs. Here, we focused on a small unit of water networks including three- to six-membered rings. The interactions between water molecules are commonly modeled by Lennard-Jones and Coulomb potentials [58]. The interaction potential energy between water molecules (a and b) is calculated by the equation:

$$v\left(a,b\right)=\;\overset{on\;a}{\underset{i}{\sum }}\overset{on\;b}{\underset{j}{\sum }}\frac{q_{i}q_{j}e^{2}}{r_{ij}}+\frac{A}{r_{oo}^{\;12}}-\frac{C}{r_{oo}^{\;6}}$$

The electrostatic attraction is expressed as the Coulombic force between two charges, *q_i_* and *q_j_*, located at a distance of *r_ij_*. The van der Waals interaction is expressed using parameters, *A* and *C*. The distance between oxygen atoms is indicated by *r_oo_*. Parameters *A* and *C* were selected to produce reasonable structural and energetic results for liquid water. Parameter values are as follows. *A* = 582,000 kcal Å^12^ mol^−1^, *C* = 595 kcal Å^6^ mol^-1^, *q_i_* = 0.834e, and *q_j_* = 0.417e. The hydrogen bond between water molecules was determined using the energy criteria of −2.25 kcal mol^−1^, which is close to the minimum value of the potential pair energy distribution [58]. More details about TWNs are provided in our earlier studies [33,39,40,41,42].

In the TWN analysis, it was presumed that the distribution of water molecules could characterize the binding site. We retrieved the ATX crystal structure with a resolution of 1.899 Å (PDB ID 3WAX) [50] from the PDB. Although this structure was obtained from Mus musculus, it shares very high sequence identity and similarity (91.9 and 94.7%, respectively) with human ATX structures (PDB ID 4ZGA) [59]. As shown in Figure S1 of the Supplementary Materials, the majority of amino acid residues within their binding sites are identical. Accordingly, MD simulation was performed on PDB ID 3WAX in the apo state for TWN analysis. After the simulation, 200 trajectories were obtained from the stable region of the root mean square deviation (RMSD) plot (Figure S2 of Supplementary Materials). Reasonably stabilized RMSD curve during 3–5 ns suggested that 200 extracted trajectories were suitable for further analysis. Water molecules present within 25 Å from the binding site were extracted for the TWN analysis. PF-8380 is a potent ATX inhibitor whose binding mode is known [45]. This compound was placed inside the binding site of ATX, and TWNs were analyzed around it.

### 3.4. Molecular Docking

Molecular docking studies were performed on the same ATX structure (PDB ID 3WAX) [50] that was used for the TWN analysis. Prior to docking, protein preparation was carried out through the previously discussed process. Compounds were built and optimized using the prepared ligand protocol. Momany–Rone partial charges [60] were applied to the protein and ligand structures. Energy minimization was performed using the CHARMM force field [61]. The CDOCKER protocol [62] of Discovery Studio 2018 (BIOVIA, San Diego, CA, USA) was used for docking. The original ligand of the crystal structure was considered for defining the binding site. Volume of the binding site was found to be 387.25 Å^3^. A simulated annealing process was performed with 2000 heating steps for the target temperature, 700 K and 5000 cooling steps for the cooling target temperature, 300 K. Then, CDOCKER energy was obtained. Finally, the binding mode of the ligands was carefully selected based on the protein–ligand interaction.

### 3.5. Synthesis and Characterization

#### 3.5.1. General Information

Solvents, reagents and starting materials were purchased from the commercial supplier. All reaction procedures were monitored by thin layer chromatography (TLC). The products were confirmed by ^1^H-NMR and ^13^C-NMR spectra. They were detected using the AVANVE Ⅲ 600 spectrometer (^1^H = 600 MHz, ^13^C = 150 MHz). Compounds were dissolved in deuterated dimethylsulfoxide (DMSO-*d*_6_) and chemical shifts were presented in ppm (d) relative to TMS as internal standard. ^1^H-NMR spectra for compounds **1**–**4** are provided in Figures S3–S6 of the Supplementary Materials, respectively. ^13^C-NMR spectra for compounds **1**–**4** are provided in Figures S7–S10 of the Supplementary Materials, respectively. High resolution mass spectra (HRMS) were obtained by AB SCIEX TripleTOF^®^ 5600 plus system (Seoul, Korea). HRMS-TOF scan results for the compounds are given in Table S1 of Supplementary Materials.

#### 3.5.2. (3,5-dichlorophenyl)methyl 4-{[4-(sulfamoylamino)phenyl]carbamoyl}piperidine-1-carboxylate (Compound **1**)

1-{[(3,5-dichlorophenyl)methoxy]carbonyl}piperidine-4-carboxylic acid (**1-a**) (0.050 g, 0.15 mmol), N-(4-aminophenyl)sulfamide (**1-b**) (0.033 g, 0.15 mmol) and 1-hydroxybenzotriazole (HOBt, 0.030 g, 0.23 mmol) were dissolved in *N*,*N*-Dimethylformamide (DMF, 2 mL). The reaction mixture was cooled to 0 °C and triethylamine (63 µL, 0.45 mmol) and (3-Dimethylaminopropyl)-N′-ethylcarbodiimide hydrochloride (EDC, 0.043 g, 0.23 mmol) were added. The reaction mixture was stirred at room temperature for 20 h. The reaction was quenched by adding ice/water and the resulting solid was filtered, washed with water and triturated with ether to afford the title compound as a white solid (0.068 g, 90%).

^1^H NMR (600 MHz, DMSO-*d*_6_) δ ppm: 9.8 (s, 1H), 9.25 (s, 1H), 7.57 (t, 1H, *J* = 1.9), 7.48 (d, 2H, *J* = 8.8), 7.43 (d, 2H, *J* = 1.7), 7.08 (d, 2H, *J* = 8.8), 6.96 (s, 2H), 5.09 (m, 2H), 4.06 (d, 2H, *J* = 13), 2.93 (br.s, 1H), 2.84 (br.s, 1H), 2.53 (m, 1H), 1.8 (d, 2H, *J* = 10.9), 1.52 (m, 2H); ^13^C NMR (150 MHz, DMSO-*d*_6_) δ 172.95, 162.41, 154.53, 141.84, 135.20, 134.63, 134.53, 131.31, 126.56, 127.88, 126.63, 120.32, 120.21, 119.61, 65.14, 43.58, 42.75, 40.53, 31.15, 28.56; HRESIMS (negative) *m*/*z* 499.0639 [M − H]^−^ (calcd 499.0615).

#### 3.5.3. (3,5-dichlorophenyl)methyl 4-[4-(sulfamoylamino)benzamido]piperidine-1-carboxylate (Compound **2**)

4-(Boc-amino)benzoic acid (**2-a**) (434 mg, 1.83 mmol), (3,5-Dichlorophenyl)methyl 4-aminopiperidine-1-carboxylate (**2-b**) (555 mg, 1.83 mmol) and 1-hydroxybenzotriazole (HOBt, 297 mg, 2.20 mmol) were dissolved in DMF (5 mL). The reaction mixture was cooled to 0 °C and triethylamine (0.3 mL, 2.2 mmol) and (3-dimethylaminopropyl)-*N*′-ethylcarbodiimide hydrochloride (EDC, 421 mg, 2.20 mmol) were added. The reaction mixture was stirred at room temperature for 16 h. The reaction was quenched by adding ice/water and the resulting solid was filtered, then triturated with ether to afford the (3,5-dichlorophenyl)methyl 4-(4-{[(tert-butoxy)carbonyl]amino}benzamido)piperidine-1-carboxylate (**2-c**) as light yellow solid (883 mg, 92%). Next, 4N HCl in dioxane solution (4 mL) was added to an ice-cooled solution of **2-c** (882 mg, 1.69 mmol) in dichloromethane (4 mL). The reaction mixture was stirred at room temperature for 4 h. The solvent was removed under reduced pressure. The resulting residue was dissolved in saturated sodium bicarbonate aqueous solution (30 mL) and extracted with ethyl acetate (30 mL × 3). The organic layer was collected, dried over anhydrous sodium sulfate, filtered and concentrated under reduced pressure to afford the (3,5-dichlorophenyl)methyl 4-(4-aminobenzamido)piperidine-1-carboxylate (**2-d**) as light yellow solid (710 mg, 99%). Formic acid (0.13 mL) was added to chlorosulfonyl isocyante (500 mg, 3.53 mmol) slowly at 0 °C and the mixture was stirred at room temperature for 2 h. The reaction mixture was concentrated under reduced pressure and the resulting solid (400 mg) was added to an ice-cooled solution of **2-d** (30 mg, 0.071 mmol) and *N*,*N*-diisopropylethylamine (25 μL, 0.14 mmol) in DMF (1 mL). The reaction mixture was stirred at room temperature for 18 h, then it was diluted with ethyl acetate (20 mL) and washed with water (20 mL × 5). The organic layer was dried over anhydrous sodium sulfate, filtered and concentrated under reduced pressure. The resulting residue was purified by column chromatography (eluent = methanol:dichloromethane = 2:98 → 5:95) to afford the title compound as white solid (23 mg, 64%)

^1^H NMR (600 MHz, DMSO-*d*_6_) δ ppm: 9.87 (s, 1H), 8.1 (d, 1H, *J* = 7.9), 7.76 (d, 2H, *J* = 8.6), 7.58 (s, 1H), 7.43 (s, 2H), 7.25 (s, 2H), 7.16 (d, 2H, *J* = 8.8), 5.09 (s, 2H), 4.01 (d, 3H, *J* = 13.6), 3.02 (br.s, 1H), 2.92 (br.s, 1H), 1.82 (d, 2H, *J* = 14.5), 1.44 (m, 2H); ^13^C NMR (150 MHz, DMSO-*d*_6_) δ 40.52, 43.28, 46.69, 62.57, 65.13, 72.77, 116.74, 120.08, 121.85, 126.64, 127.91, 128.69, 134.55, 141.84, 142.60, 152.61, 154.56, 165.61, 166.85, 188.74; HRESIMS (negative) *m*/*z* 499.0612 [M − H]^−^ (calcd 499.0615).

#### 3.5.4. (3,5-dichlorophenyl)methyl 5-{[4-(sulfamoylamino)phenyl]carbamoyl}-2,3-dihydro-1H-isoindole-2-carboxylate (Compound **3**)

To an ice-cooled solution of N-(4-aminophenyl)sulfamide (**1-a**) (14 mg, 0.066 mmol) in DMF (1 mL) and 2-{[(3,5-dichlorophenyl)methoxy]carbonyl}-2,3-dihydro-1*H*-isoindole-5-carboxylic acid (**1-c**) (30 mg, 0.082 mmol), *N*,*N*-diisoprolyethylamine (0.07 mL, 0.41 mmol) and benzotriazol-1-yl-oxytripyrrolidinophosphonium hexafluorophosphate (PyBOP, 64 mg, 0.12 mmol) were added. The reaction mixture was stirred at room temperature for 15 h under nitrogen atmosphere. After completion, water (15 mL) was added to the reaction mixture and it was extracted with ethyl acetate (30 mL × 3). The organic layer was washed with brine, dried over anhydrous sodium sulfate, filtered, and concentrated under reduced pressure. The resulting residue was triturated with diethyl ether and methanol to afford the title compound as white solid (20 mg, 57%).

^1^H NMR (600 MHz, DMSO-*d*_6_) δ ppm: 10.17 (d, 1H, *J* = 4.8), 9.35 (s, 1H), 7.91 (m, 2H), 7.66 (d, 2H, *J* = 8.4), 7.58 (d, 1H, *J* = 1.7), 7.5 (m, 3H), 7.15 (d, 2H, *J* = 8.8), 7.02 (s, 2H), 5.18 (d, 2H, *J* = 3.7), 4.82 (br.s, 2H), 4.74 (br.s, 2H); ^13^C NMR(150 MHz, DMSO-*d*_6_) δ 40.52, 51.99, 52.12, 52.56, 52.69, 65.13, 65.17, 119.31, 121.63, 122.66, 122.80, 123.23, 123.33, 126.57, 126.62, 127.47, 127.89, 134.32, 134.56, 135.78, 141.76, 141.80, 154.15; HRESIMS (negative) *m*/*z* 533.0469 [M − H]^−^ (calcd 533.0459).

#### 3.5.5. (3,5-dichlorophenyl)methyl 5-[4-(sulfamoylamino)benzamido]-2,3-dihydro-1*H*-isoindole-2-carboxylate (Compound **4**)

To an ice-cooled solution of 4-(sulfamoylamino)benzoic acid (**3-a**) (23 mg, 0.106 mmol) in DMF (1 mL) and (3,5-dichlorophenyl)methyl 5-amino-2,3-dihydro-1H-isoindole-2-carboxylate (**3-b**) (30 mg, 0.090 mmol), *N*,*N*-diisoprolyethylamine (0.05 mL, 0.31 mmol) and PyBOP (70 mg, 0.135 mmol) were added. The reaction mixture was stirred at room temperature for 15 h under nitrogen atmosphere. After completion, the reaction mixture was diluted with ethyl acetate (20 mL) and washed with water (15 mL × 4). The organic layer was washed with brine, dried over anhydrous sodium sulfate, filtered and concentrated under reduced pressure. The resulting residue was triturated with diethyl ether and n-hexane to afford the title compound as beige solid (17 mg, 35%).

^1^H NMR (600 MHz, DMSO-*d*_6_) δ ppm: 10.12 (s, 1H), 9.98 (s, 1H), 7.9 (d, 2H, *J* = 8.1), 7.8 (d, 1H, *J* = 13), 7.63 (d, 1H, *J* = 8.1), 7.57 (s, 1H), 7.5 (s, 2H), 7.31 (m, 3H), 7.24 (d, 2H, *J* = 8.8), 5.16 (s, 2H), 4.76 (s, 1H), 4.72 (s, 1H), 4.67 (s, 1H), 4.63 (s, 1H); ^13^C NMR(150 MHz, DMSO-*d*_6_) δ 40.52, 51.88, 52.26, 52.47, 52.85, 65.09, 116.78, 120.28, 123.37, 126.59, 126.62, 127.87, 129.17, 131.55, 132.13, 134.56, 137.03, 137.66, 139.19, 141.83, 143.09, 154.15, 165.42; HRESIMS (negative) *m*/*z* 533.0468 [M − H]^−^ (calcd 533.0459).

### 3.6. Biological Experiment

#### 3.6.1. In Vitro bis-pNPP Assay

We confirmed the ATX inhibitory activity of each compound using bis-pNPP assay. First, 50 μL of 60 mM Tris-HCl (pH 9.0) solution was poured into a 96-well plate and 10 μL of the test compound solution (10% dimethyl sulfoxide) was added. Subsequently, 20 μL of a 3.75 nM human ENPP2 solution (buffer: 50 mM Tris-HCl (pH 8.5), 5 mM CaCl_2_, 2.5 mM MgCl_2_, 0.002% Brij 35) and 20 μL of 25 mM bis-pNPP solution (buffer: 50 mM Tris-HCl (pH 9.0)) were added and mixed. Spectrophotometric measurement was carried out at 405 nm at intervals of 10 s while reacting under 37 °C for 10 min.

#### 3.6.2. Ex Vivo Human Plasma Assay

First, 5 μL of each test compound solution (10 mM, 100% dimethyl sulfoxide) was diluted 100 times with 495 μL of methanol and then 3 μL of a diluted methanol solution (1% dimethyl sulfoxide) was added to a human or rat serum solution. Next, 12 μL of the test compound solution was mixed into 48 μL of 100% serum in order to make 5 concentrations, and each tube was placed in a constant temperature water bath at 37 °C for 15 min. Subsequently, 10 mg/mL 18: 1 LPC solution (50% ethanol) was diluted to 375 μg/mL with serum and 2 μL was dispensed into each tube to make 50 μL. Each tube was reacted in a constant temperature water bath at 37 °C for 3 h. Then, 100 μL of a 0.5 μM 17:0 LPA solution (Chloroform/Methanol/Water = 65/35/8) was added to the reaction tube. Reaction mixture was centrifuged at 14,000 rpm, at 4 °C for 10 min in a centrifuge. Thereafter, 100 μL of 50% methanol solution was first dissolved in a 96-well polypropylene plate (Agilent Technologies 5042-1385, Santa Clara, CA, USA), and then 50 μL of the supernatant was carefully transferred from the centrifuged tube to the plate. Then, it was covered with a well cap (Thermo 276011, Thermo Fisher Scientific Solutions LLC, Seoul, Korea) and analyzed by LC-MS/MS (Agilent 1260). IC_50_ values were calculated using the Grafit5 software after calculating the % inhibition values by the formula: 100 − (3 h serum + test solution/3 h serum + control group) × 100.

## 4. Conclusions

In continuation with our previous efforts to develop potent ATX inhibitors, we have reported novel ATX inhibitors in the present study. We used an in-house computational method to analyze the water networks in the binding site of ATX. This analysis led to the identification of a pharmacophoric site in the binding pocket. We designed new compounds considering the pharmacophoric site. They showed high inhibitory activities against ATX.

## Figures and Tables

**Table 1:** Structures, activities, and predicted physicochemical properties of the compounds.

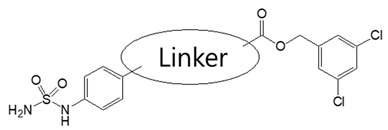					
Compound	Linker	Bis-pNPP IC_50_ (nM)	MW	ClogP ^a^	PSA ^b^
**1**	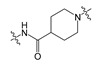	250.00	501.38	3.59	139.21
**2**	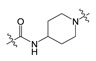	44.50	501.38	3.63	139.21
**3**	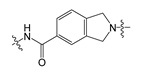	3.86	535.40	4.36	139.21
**4**	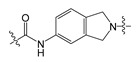	12.70	535.40	4.70	139.21

^a^ calculated by Chemdraw, ^b^ calculated by Discovery Studio 2018.

## Supplementary Materials

The following are available online, Figure S1: Sequence alignment of Mus musculus autotaxin (PDB ID 3WAX) and human autotaxin (PDB ID entry code: 4ZGA). They have high similarity with 91.9% and 94.7%, sequence identity and similarity, respectively. Residues near binding site are marked in the red boxes. Most of the marked amino acid residues are identical, Figure S2: RMSD plot for the alpha-carbon atoms of the ATX during 10 ns MD simulation. The 200 trajectories were extracted from the stable region (3–5 ns, marked by the blue box) for the TWN analysis, Figure S3: ^1^H-NMR (600 MHz, DMSO-*d*_6_) spectra of compound **1**, Figure S4: ^1^H-NMR (600 MHz, DMSO-*d*_6_) spectra of compound **2**, Figure S5: ^1^H-NMR (600 MHz, DMSO-*d*_6_) spectra of compound **3**, Figure S6: ^1^H-NMR (600 MHz, DMSO-*d*_6_) spectra of compound **4**, Figure S7: ^13^C-NMR (150 MHz, DMSO-*d*_6_) spectra of compound **1**, Figure S8: ^13^C-NMR (150 MHz, DMSO-*d*_6_) spectra of compound **2**, Figure S9: ^13^C-NMR (150 MHz, DMSO-*d*_6_) spectra of compound **3**, Figure S10: ^13^C-NMR (150 MHz, DMSO-*d*_6_) spectra of compound **4**, Table S1: HRMS-TOF scan result (Triple TOF 5600 plus system (AB SCIEX)).
