# Supplementary material for: Pharmacophoric Site Identification and Inhibitor Design for Autotaxin
Source: Molecules. 2019 Aug 1;24(15):2808. doi: 10.3390/molecules24152808 (PMC6696049; doi:10.3390/molecules24152808)
Supplement: Supplementary file 1 [file molecules-24-02808-s001.pdf]

Supplementary Materials

Pharmacophoric Site Identification and Inhibitor Design for Autotaxin

Myeong Hwi Lee [1], Dae-Yon Lee [2], Anand Balupuri [1], Jong-Woo Jeong [1] and Nam Sook Kang [1,*]

[1] Graduate School of New Drug Discovery and Development, Chungnam National University, 99 Daehak-ro, Yuseong-gu, Daejeon 34134, Korea

[2] LegoChem Biosciences, Inc., 8-26 Munoyeongseo-ro, Daedeok-gu, Daejeon, 34302, Republic of Korea

[*] Correspondence: nskang@cnu.ac.kr; Tel.: +82-42-821-8626

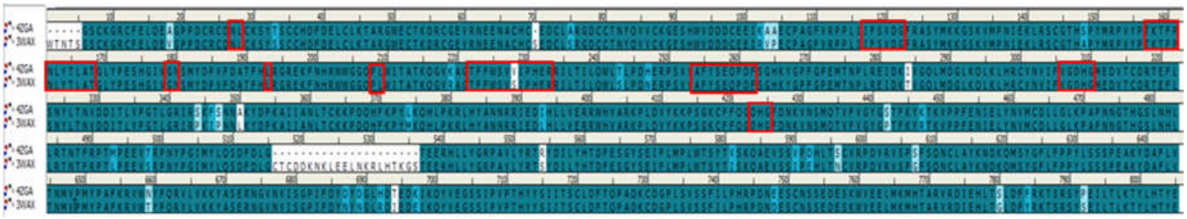

Figure S1. Sequence alignment of Mus musculus autotaxin (PDB ID 3WAX) and human autotaxin (PDB ID entry code: 4ZGA). They have high similarity with 91.9% and 94.7%, sequence identity and similarity, respectively. Residues near binding site is marked in the red boxes. Most of the marked amino acid residues are identical.

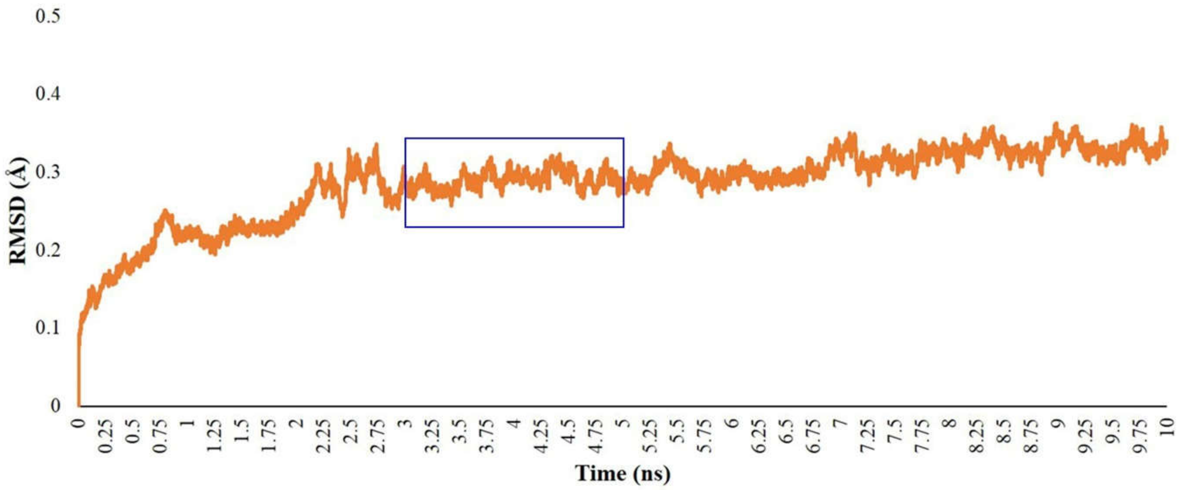

Figure S2. RMSD plot for the alpha-carbon atoms of the ATX during 10 ns MD simulation. The 200 trajectories were extracted from the stable region (3-5 ns, marked by blue box) for the TWN analysis.

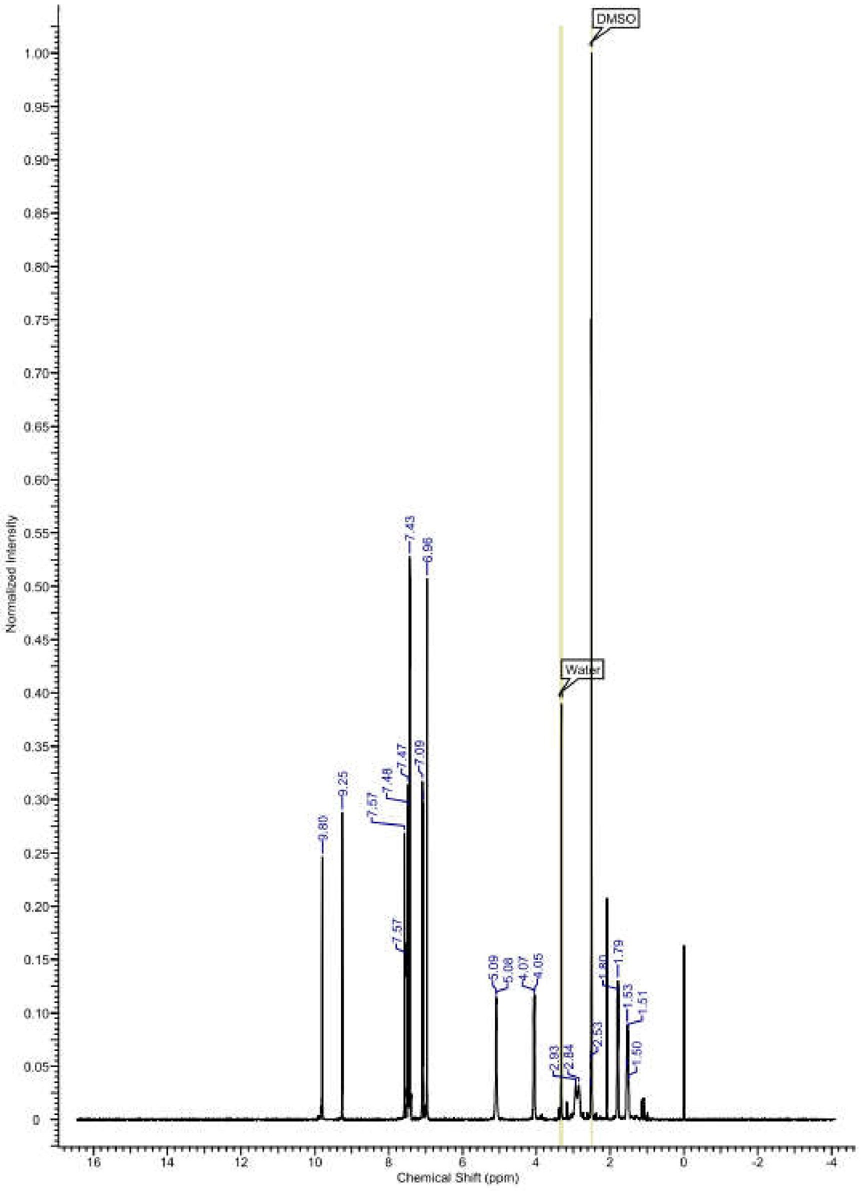

Figure S3. ^{1}H-NMR (600 MHz, DMSO-d_6) spectra of compound **1**.

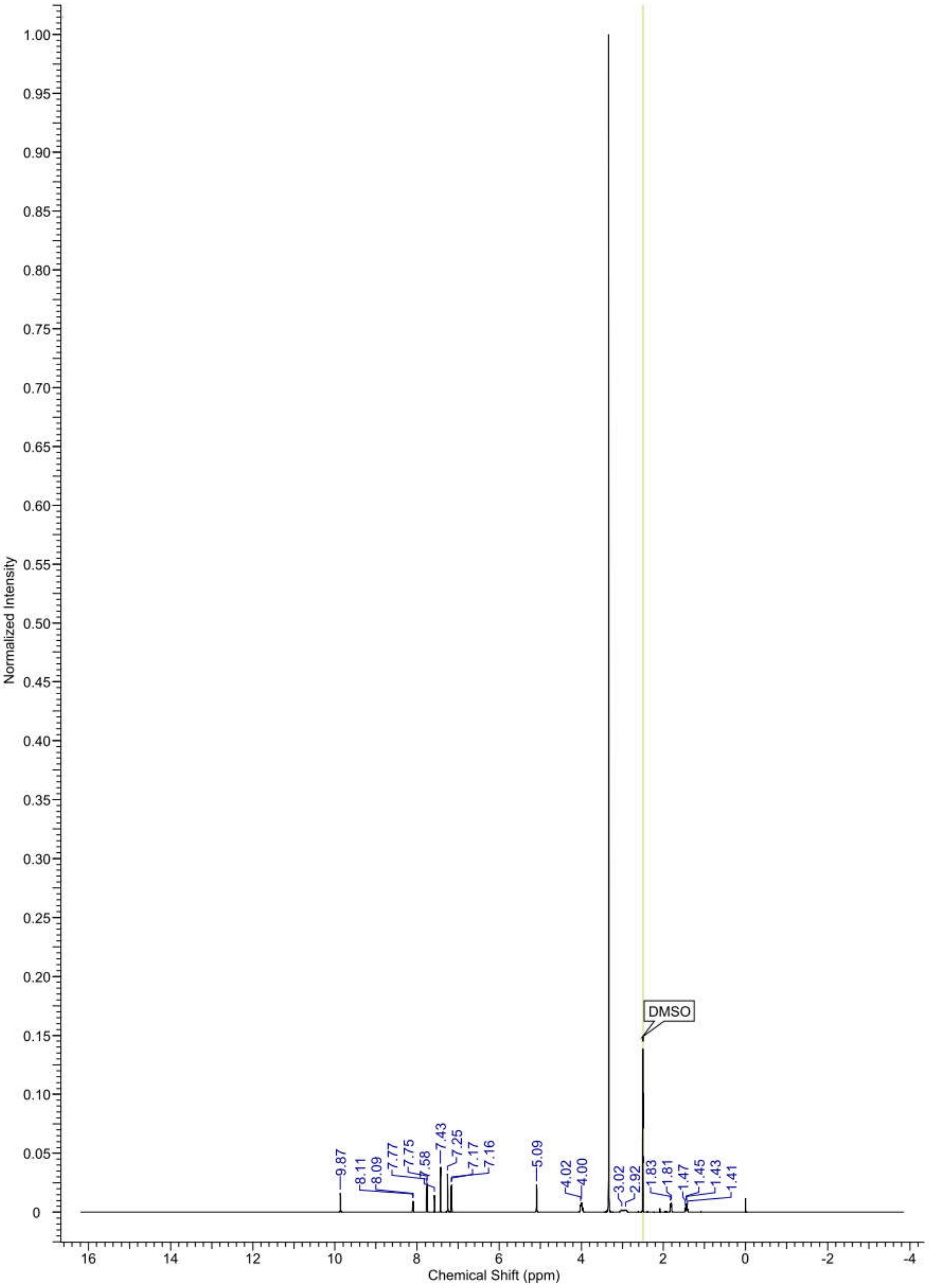

Figure S4. ^{1}H-NMR (600 MHz, DMSO-d$_6$) spectra of compound **2**.

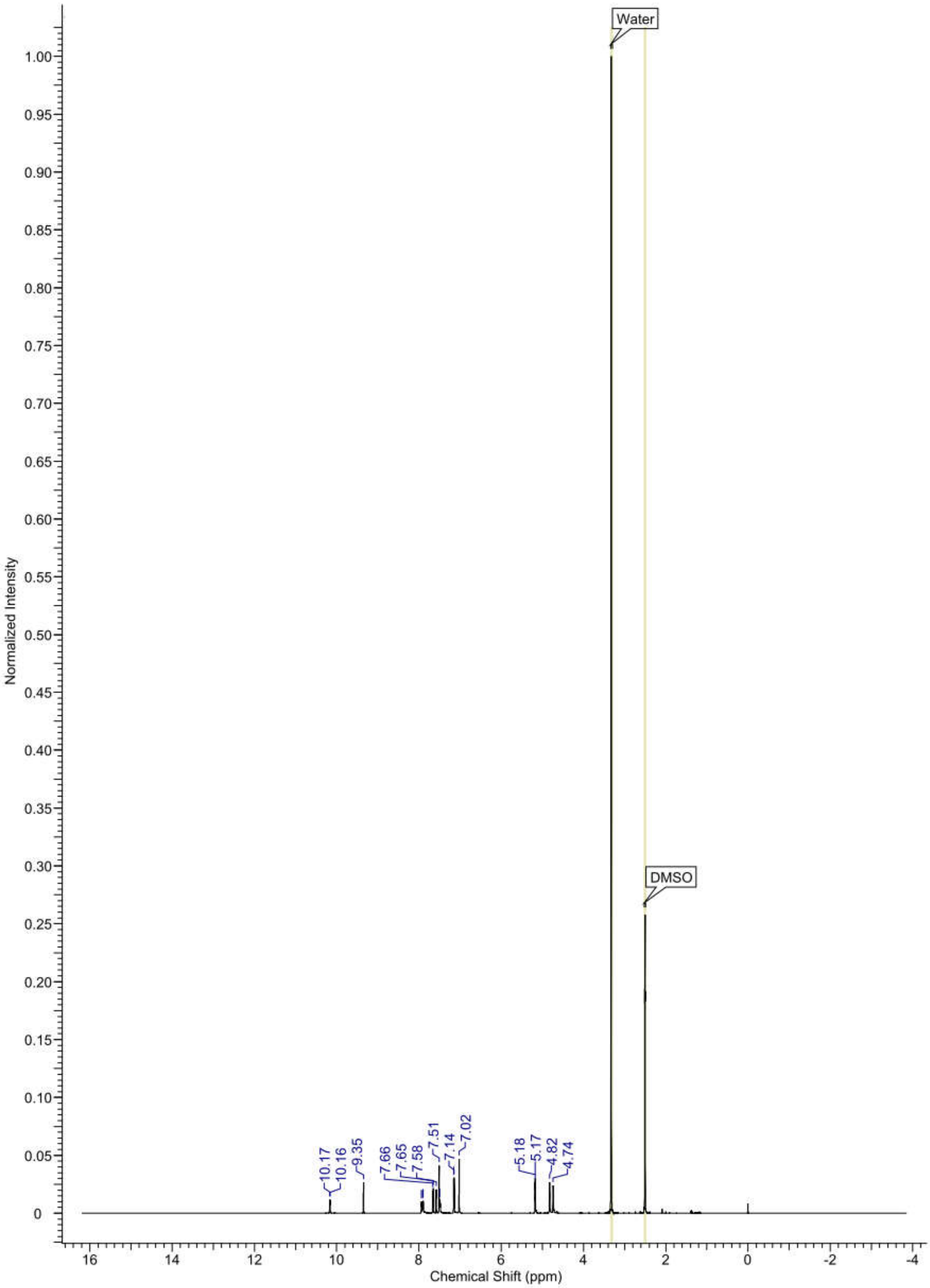

Figure S5. ^{1}H-NMR (600 MHz, DMSO-d6) spectra of compound **3**.

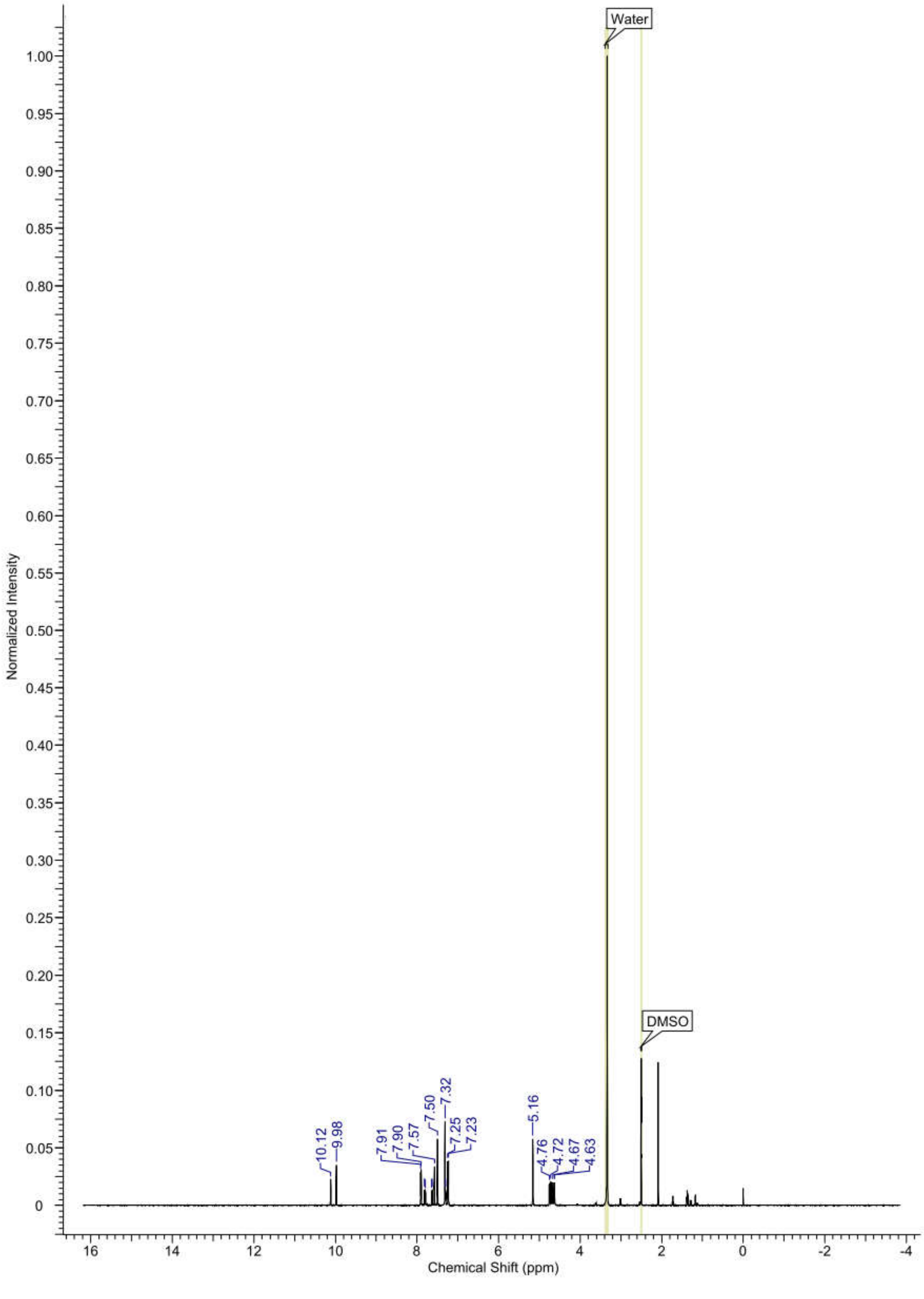

Figure S6. ^{1}H-NMR (600 MHz, DMSO-d_6) spectra of compound **4**.

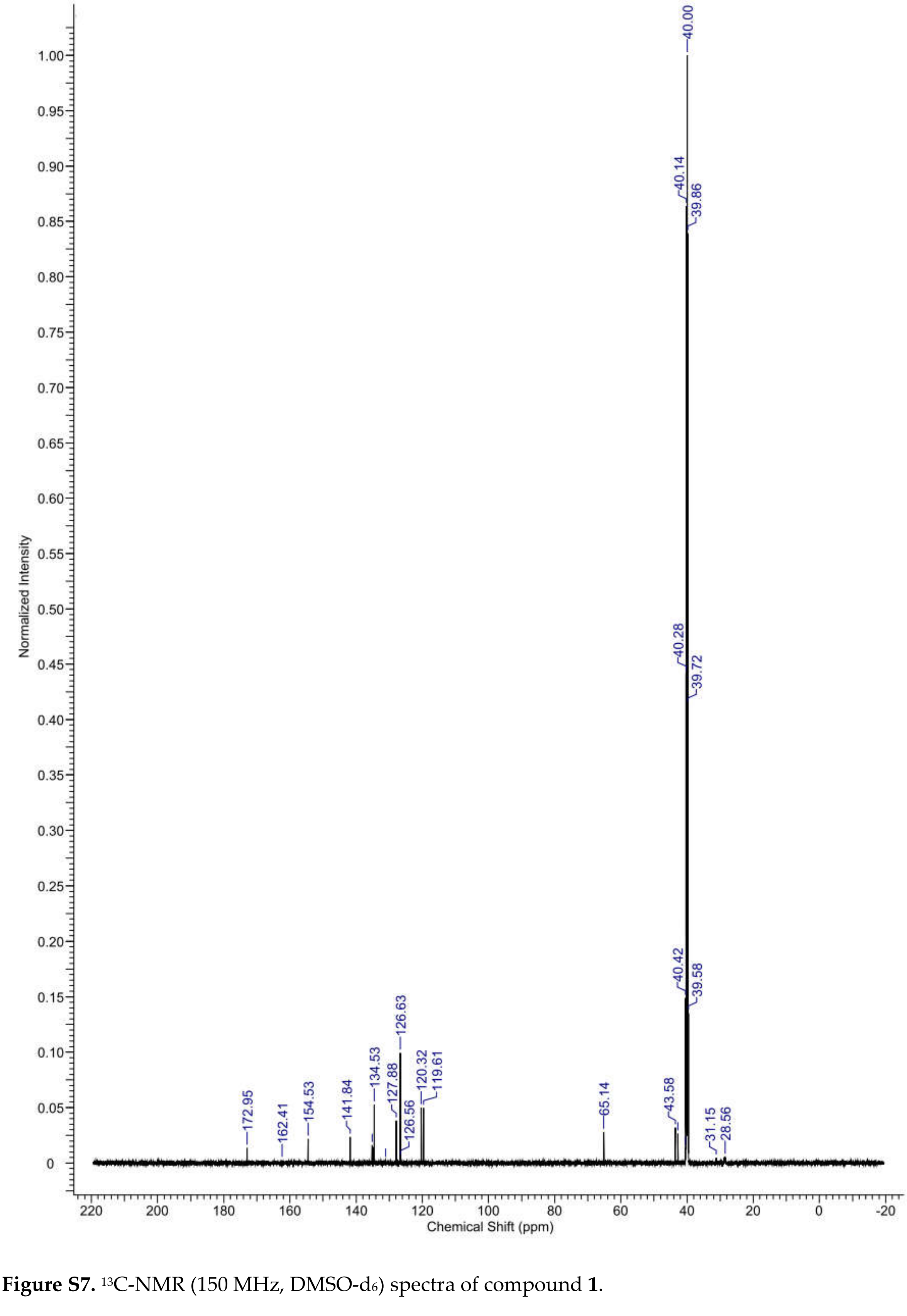

Figure S7. ^{13}C-NMR (150 MHz, DMSO-d_6) spectra of compound **1**.

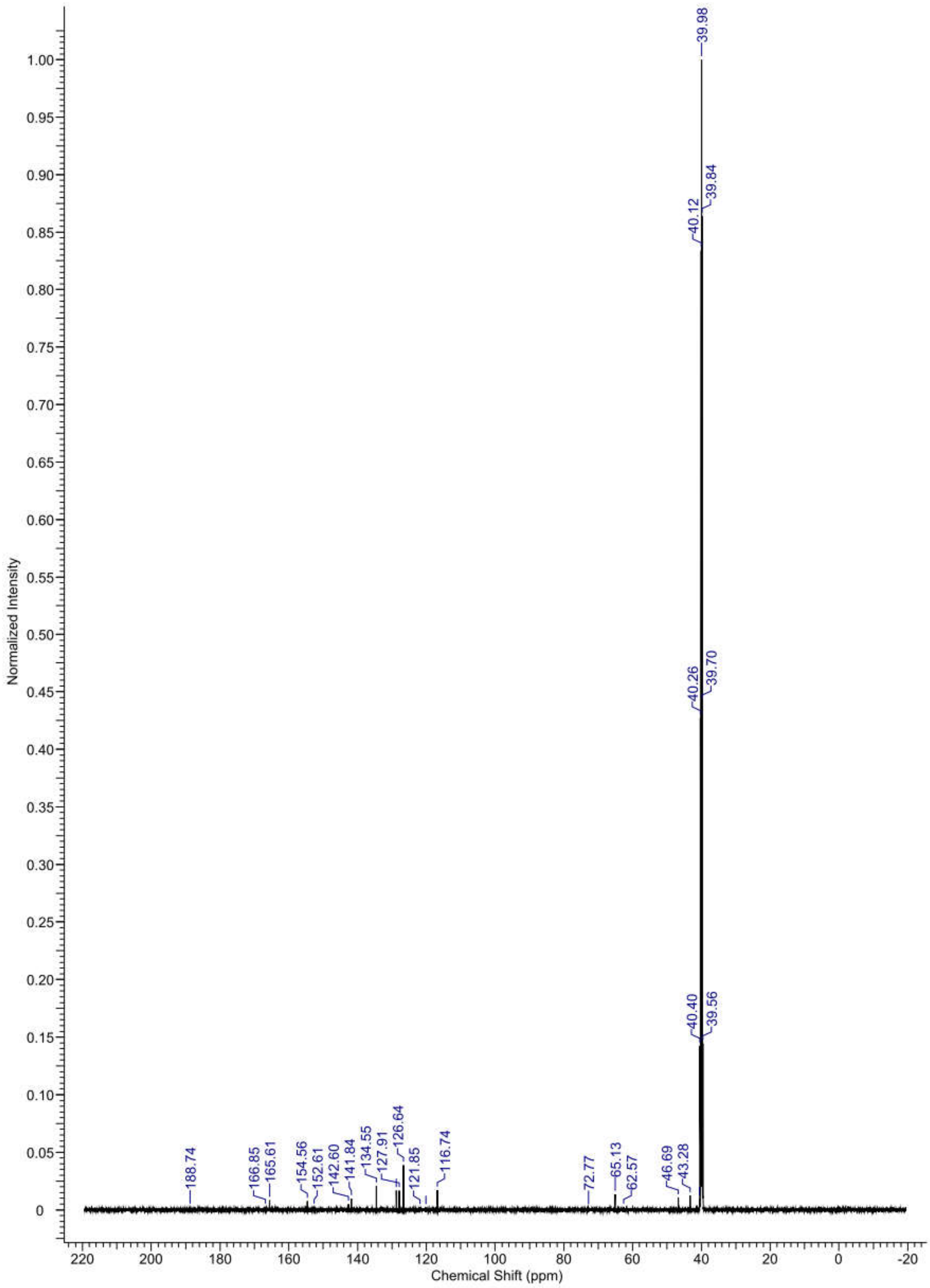

Figure S8. ^{13}C-NMR (150 MHz, DMSO-d_6) spectra of compound **2**.

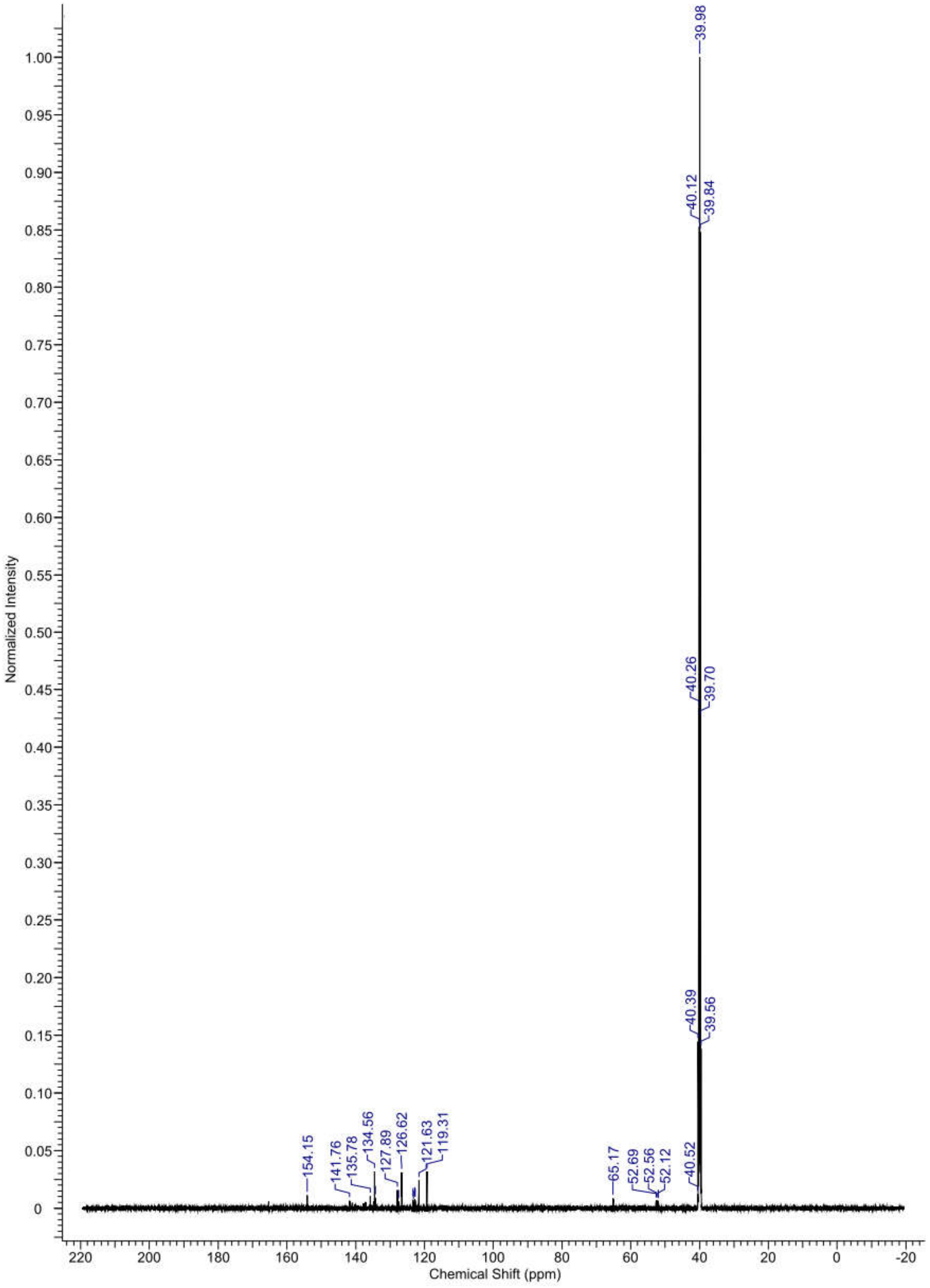

Figure S9. ^{13}C-NMR (150 MHz, DMSO-d_6) spectra of compound **3**.

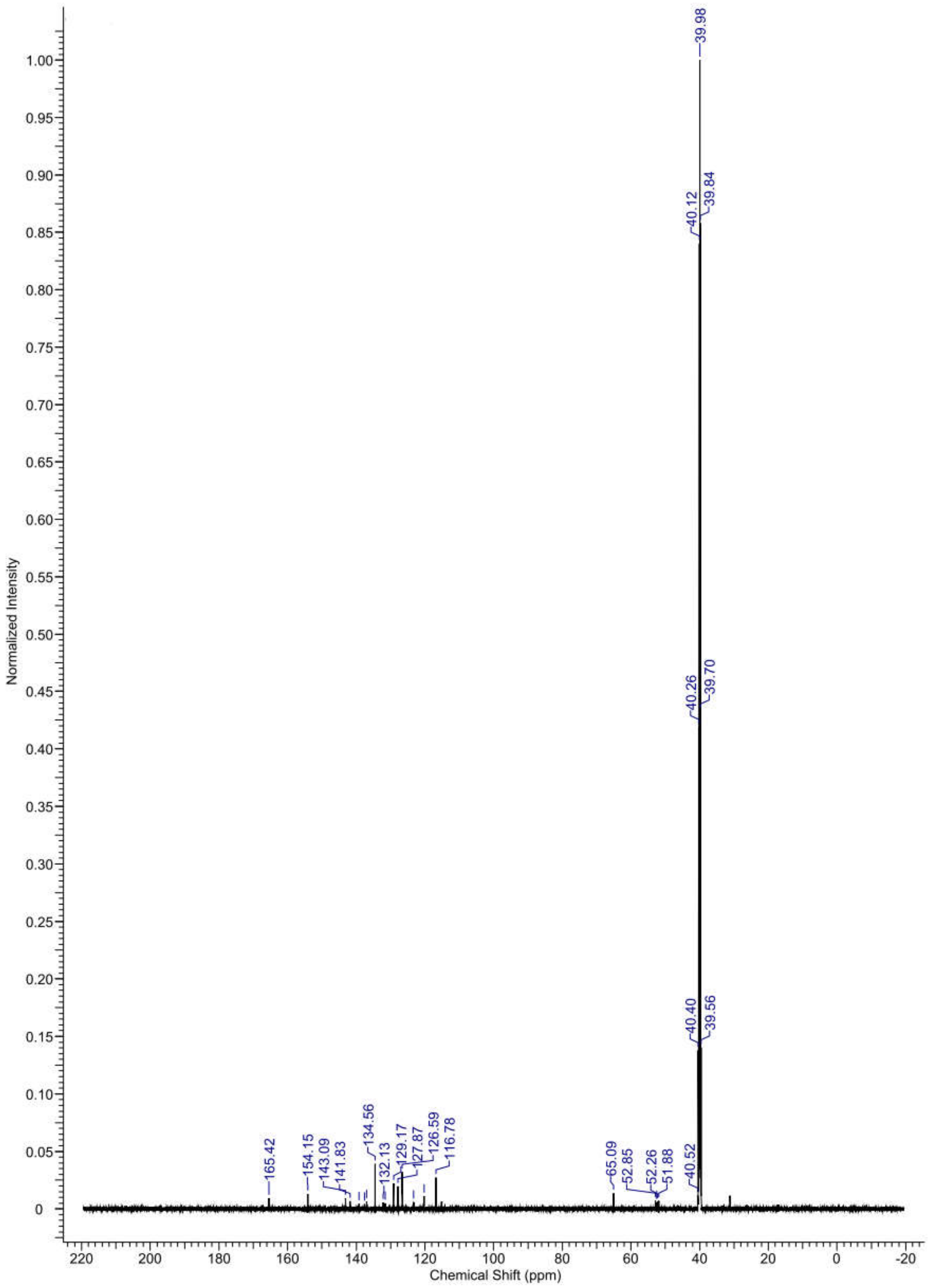

Figure S10. ^{13}C-NMR (150 MHz, DMSO-d_6) spectra of compound **4**.

Table S1. HRMS-TOF scan result (Triple TOF 5600 plus system (AB SCIEX)).

ESI (-)	Calculated (m/z)	Observed (m/z)	Mass shift (Da)	Mass Accuracy (ppm)
Compound **1**	499.0615	499.0639	0.002	4.77
Compound **2**	499.0615	499.0612	0.000	0.60
Compound **3**	533.0459	533.0469	0.001	1.88
Compound **4**	533.0459	533.0468	0.001	1.69
Criteria				< 10

*Information

-Scan type: Electrospray ionization (ESI), negative TOF MS mode

-Scan range: 100 to 1,000 Da

-Infusion rate: 3 μL/min
